# First-Time Presence of African Swine Fever Virus Genotype II in Nigeria

**DOI:** 10.1128/MRA.00350-21

**Published:** 2021-07-01

**Authors:** Adeyinka J. Adedeji, Pam D. Luka, Rebecca B. Atai, Toyin A. Olubade, Dupe A. Hambolu, Mary A. Ogunleye, Vincent B. Muwanika, Charles Masembe

**Affiliations:** a National Veterinary Research Institute, Vom, Nigeria; b Federal Department of Veterinary and Pest Control Services, Lagos, Nigeria; c Ministry of Agriculture, Ikeja, Lagos State, Nigeria; d College of Agricultural & Environmental Sciences, Makerere University, Kampala, Uganda; e College of Natural Sciences, Makerere University, Kampala, Uganda; Queens College CUNY

## Abstract

A confirmed African swine fever (ASF) outbreak in Nigeria was further investigated by partial sequencing of the *B464L* and *E183L* genes of ASF virus (ASFV). Results revealed the first-time presence of ASFV genotype II in Nigeria and West Africa. This finding has serious implications for control measures and food security.

## ANNOUNCEMENT

African swine fever (ASF) is a highly fatal disease of pigs caused by ASF virus (ASFV), a double-stranded DNA virus of the genus *Asfivirus* and family *Asfarviridae* (1). Twenty-four genotypes of ASFV have been identified based on partial sequencing of the genes *B646L*, encoding the p72 capsid protein, and *E183L*, encoding the p54 envelope protein (1, 2). Only genotype 1 is known to be circulating in Nigeria and West Africa, while ASFV genotype II circulates in Europe and Asia (1, 3–5). In this study, investigation of an ASF outbreak that occurred in May 2020 on a pig farm in Oko Oba, Lagos, Nigeria, was carried out by characterizing the *B646L* (p72) and *E183L* (p54) genes of ASFV. Tissue samples (liver and spleen) were collected from dead pigs that showed clinical signs of ASF, and DNA was extracted using the QIAamp DNA minikit (Qiagen, Hilden, Germany).

**FIG 1:**
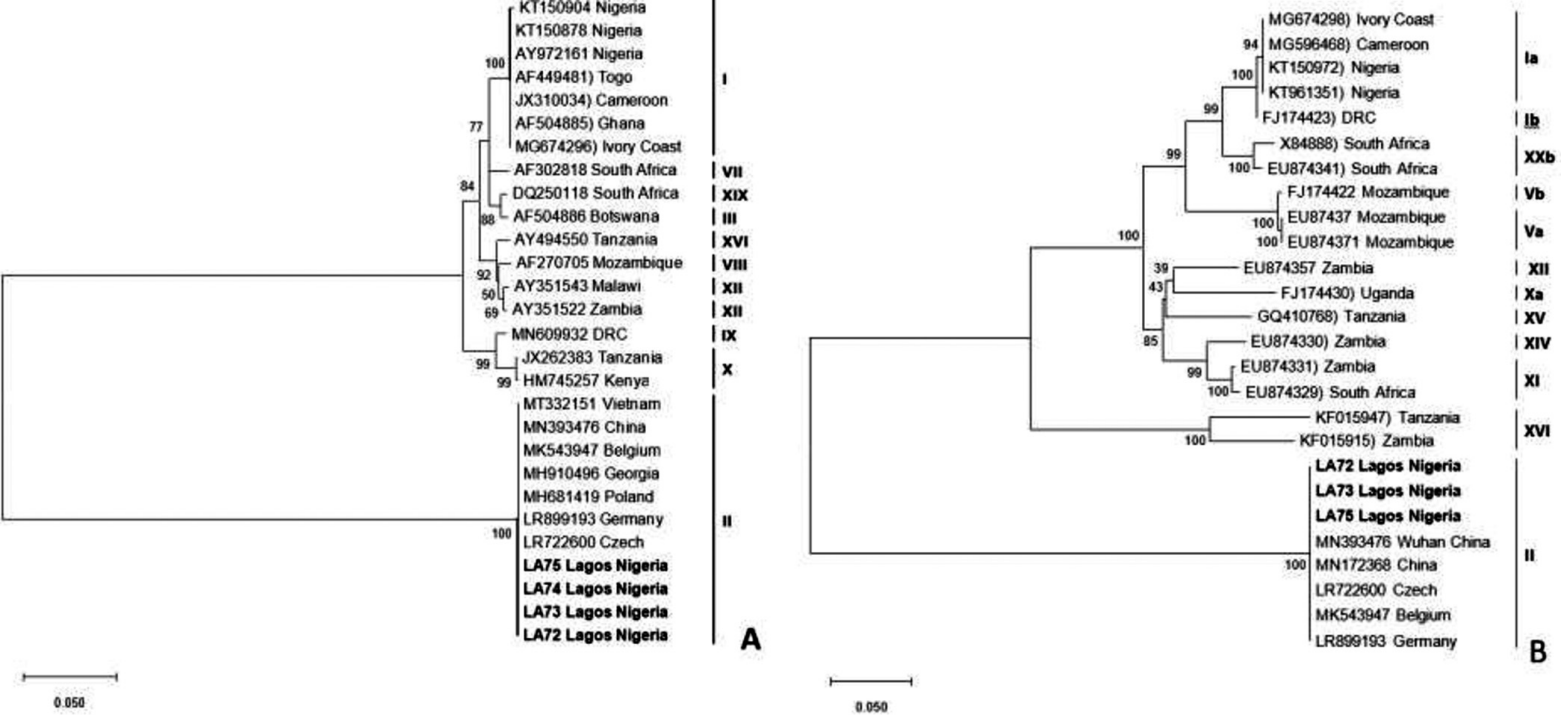
(A) Phylogenetic tree of partial *B646L* (p72) gene nucleotide sequences of Nigerian ASFV from a pig farm in Lagos, Nigeria. The tree was inferred using the minimum evolution method with 1,000 bootstrap replicates. The Nigerian ASFV isolates from this study are indicated in boldface and clustered with ASFV genotype II. (B) Phylogenetic tree based on the full-length *E183L* gene (p54). The phylogenetic tree was inferred using the minimum evolution method. The Nigerian ASFV sequences analyzed in this study are indicated by boldface and clustered within genotype II. The scale bar indicates the number of nucleotide substitutions per site.

ASFV was detected in all tissue samples (4/4) collected from the pig farm using real-time PCR and analyzed at the National Veterinary Research Institute, Vom, Nigeria, as previously described (6). Genotyping was carried out by sequencing the *B646L* gene, which encodes the p72 protein, and *E183L* gene, which encodes the p54 protein, as previously described (7). The sequencing of the 4 ASFV-positive samples was carried out by the Sanger sequencing method at Macrogen Inc. (Netherlands). Chromatogram editing, assembly, and alignment of the raw sequences were done using the Staden package (http://staden.sourceforge.net/) and Bioedit (8). Two ASFV genes were sequenced, namely, *B464L* and *E183L*, but 7 sequences were obtained, including 4 sequences for *B464L* and 3 for *E183L.* They were compared with other sequences in the GenBank using the Basic Local Alignment Search Tool (BLAST) (https://blast.ncbi.nlm.nih.gov/Blast.cgi). Twenty-four *B646L* (p72) and 21 *E183L* (p54) sequences representing the known genotypes were retrieved from GenBank for combined phylogenetic analysis with those generated from this study using MEGA X (9). Default parameters were applied for all software used. The minimum evolution method with 1,000 bootstraps was used for phylogenetic reconstruction. The comparison with ASFV sequences in GenBank revealed a similarity of 99% to 100% with ASFV genotype II from Vietnam (GenBank accession number MT332151) and China (MN393476). Phylogenetic analysis showed that sequences of both the p72 and p54 genes strongly clustered with ASFV genotype II (Fig. 1). The clustering of the sequences in our study with other genotype II sequences from different geographical areas had high bootstrap support. All of the p72 and p54 ASFV sequences that were obtained have been deposited in GenBank. In this report, sequencing and phylogenetic analysis revealed that ASFV genotype II was responsible for an ASF outbreak on a pig farm in Nigeria. Nucleotide sequences for p72 and p54 were used to describe the ASFV genotype in this study. Sequences from previous outbreaks in Nigeria and West Africa clustered only with genotype I. For the first time, ASFV genotype II was found and reported in West Africa. Introducing a new ASFV genotype into Nigeria presents complex epidemiology and will further complicate the already constrained control measures against the disease in the region.

### Data availability.

The ASFV genome sequences in this report are available in GenBank under accession numbers MW852486 to MW852492.
